# Autism Spectrum Disorder (ASD) and Fragile X Syndrome (FXS): Two Overlapping Disorders Reviewed through Electroencephalography—What Can be Interpreted from the Available Information?

**DOI:** 10.3390/brainsci5020092

**Published:** 2015-03-27

**Authors:** Niamh Mc Devitt, Louise Gallagher, Richard B. Reilly

**Affiliations:** 1 School of Medicine, Trinity College, the University of Dublin, Dublin, Ireland; E-Mails: lgallagh@tcd.ie (L.G.); reillyri@tcd.ie (R.B.R.); 2 Trinity Centre for Bioengineering, Trinity College Dublin, the University of Dublin, Dublin, Ireland; 3 Trinity College Institute for Neuroscience, Trinity College Dublin, the University of Dublin, Dublin, Ireland; 4 Department of Psychiatry, Trinity College Dublin, the University of Dublin, Dublin, Ireland; 5 Institute of Molecular Medicine, Trinity Centre for Health Sciences, St James’ Hospital, Dublin, Ireland; 6 Linn Dara Child and Adolescent Mental Health Services, Cherry Orchard Hospital Dublin 10, Dublin, Ireland; 7 School of Engineering, Trinity College Dublin, the University of Dublin, Dublin, Ireland

**Keywords:** autism spectrum disorders, Fragile X Syndrome, electroencephalography, Event Related Potentials, resting state EEG

## Abstract

Autism Spectrum Disorder (ASD) and Fragile X syndrome (FXS) are neurodevelopmental disorders with different but potentially related neurobiological underpinnings, which exhibit significant overlap in their behavioural symptoms. FXS is a neurogenetic disorder of known cause whereas ASD is a complex genetic disorder, with both rare and common genetic risk factors and likely genetic and environmental interaction effects. A comparison of the phenotypic presentation of the two disorders may highlight those symptoms that are more likely to be under direct genetic control, for example in FXS as opposed to shared symptoms that are likely to be under the control of multiple mechanisms. This review is focused on the application and analysis of electroencephalography data (EEG) in ASD and FXS. Specifically, Event Related Potentials (ERP) and resting state studies (rEEG) studies investigating ASD and FXS cohorts are compared. This review explores the electrophysiological similarities and differences between the two disorders in addition to the potentially associated neurobiological mechanisms at play. A series of pertinent research questions which are suggested in the literature are also posed within the review.

## 1. Introduction

The identification of abnormalities in brain activity and behaviour that distinguish children with Fragile X Syndrome (FXS) from children with Autistic Spectrum Disorders (ASD), as well as from typically developing (TD) children, may provide important insights into these disorders. This may in turn lead to earlier diagnosis and treatments that are more specific to the requirements of each of these disorders.

ASD and FXS are childhood-onset neurodevelopmental disorders associated with deficits in cognition and behaviour. FXS is the most commonly observed genetic cause of ASD. FXS is a monogenetic disorder caused by a CGG expansion repeat in the 5′ UTR of the *FMR1* gene which encodes Fragile X Mental Retardation Protein (FMRP). Males and females with between 55 and 200 repeats of the CGG segment are described as having a premutation of the *FMR1* gene. More than 200 repeats result in a full mutation which gives rise to FXS. This causes hypermethylation to occur in the *FMR1* gene causing loss of expression of the protein FMRP. FMRP is a translational repressor silencing up to 4% of brain expressed mRNA, and is involved in regulating mRNA transport and protein synthesis both pre and post-synaptically. Animal knockout models of FXS have shown that loss of FMRP expression leads to elongated dendritic spines associated with an increase in long-term depression (LTD) and reduced synaptic maturation [1,2]. Increased synthesis of metabotropic glutamate receptors (mGluR) is associated with long term depression (LTD) and underpins the mGluR neurobiological theory of FXS [3]. Additionally, deficits in GABAergic signalling have been implicated [4,5]. ASDs are complex polygenetic conditions associated with both rare genetic risks (*i.e.*, copy number variants or single nucleotide variants) in a small minority of cases and more common genetic variants acting in combination with environmental risk factors [6]. Similarly to FXS, deficits in glutamatergic and GABAergic signalling have been implicated and this has become an area of increasing interest in ASD research [7,8,9].

The reported numbers of people with FXS that also have ASD varies widely across studies and estimates of the prevalence varies between 5%–60% [10,11,12,13,14,15] with additional reports that 90% of patients with FXS exhibit some type of autistic characteristic [16]. The association between ASD and FXS was first highlighted by Brown *et al.* in 1982 [17] and evidence from genetic, behavioural, post-mortem and neuroimaging studies further supports the evidence of this overlap between the disorders. Several common ASD symptoms in the behavioural domain have been reported in FXS, such as gaze avoidance [18], hand flapping, repetitive behaviours [19], and repetitive speech [20] as well as, sensory motor deficits, attention deficits, anxiety and aggression. Rogers *et al.* observed that 33% of children with FXS between 21 and 48 months could not be differentiated behaviourally from children of the same age with ASD [10]. Furthermore, studies have shown that some individuals in the FXS premutation range (between 55–200 repeats) have ASD [21]. The presence of core ASD symptoms in FXS has been linked with a greater degree of impairment relating to social interaction, cognition and adaptive behaviour compared with individuals with FXS alone [22]. Despite the similarity of clinical symptoms, however, it is not clear whether these are related at a neurobiological level. Studies comparing endophenotypes in the two disorders are rare and inconclusive. Gaze fixation in people with ASD has been found to be more atypical than in people with FXS, however FMRI activation in the Fusiform Gyrus (FG), an area associated with face processing, has been observed to be decreased in both groups in comparison to Typically Developing (TD) control subjects [23]. A higher prevalence in males compared to females is also common between the conditions, although the ratio differs. FXS is two to three times more common in males and ASD has been shown to be approximately four times more likely in males. Higher male prevalence of FXS is understandable in context of an X-linked genetic aetiology; however this has not been demonstrated for ASD to date.

Structural and functional Magnetic Resonance Imaging (MRI), as well as post-mortem studies have been widely used to investigate brain structure and function in ASD (See Table 1 for examples). MRI studies in FXS are less common given lower prevalence rates and more methodological challenges, such as difficulties tolerating MRI recordings in individuals with cognitive impairment. A limited number of neuroanatomical studies have compared brain growth abnormalities in both disorders. Hazlett *et al.* (2012), observed an increase in total brain volume (TBV) in ASD from 2–4 years [24]. Children with FXS exhibited an area-specific pattern of increased growth, particularly in the temporal lobe white matter, cerebellar gray matter, and caudate nucleus (CN). The FXS group were also shown to have significantly greater CN, Globus Pallidus (GP) and putamen volume than the ASD group. Children with FXS were generally shown to exhibit increased volume of both gray and white matter areas in contrast with controls, however the increased volumes were less robust than the increased volumes observed in the ASD group, when compared to controls. Cerebellar and CN volume changes are some of the most commonly reported and replicated abnormalities in both ASD and FXS [25], suggesting that mechanisms regulating early developmental processes appear to be disrupted in both disorders [24]. Overall volumetric abnormalities are one of the most consistent neuroanatomical findings in both ASD and FXS, particularly in early life although not exclusively. Volumetric atypicalities in adolescence and adulthood are also exhibited in ASD [26,27]. Although MRI is a very useful tool in the investigation of neurodevelopmental disorders, there are some limitations to its uses, for example, tolerability of the MRI procedure for children with more severe deficits, poor temporal resolution and expense. EEG on the other hand, is easy to use, relatively inexpensive and exhibits fine temporal resolution of brain activity.

The focus of this review are studies employing EEG-based neuroimaging rather than MRI, however findings from MRI studies may relate to EEG findings, ultimately providing a better picture of brain abnormalities in both these neurodevelopmental disorders. Buchmann *et al.* (2011) investigated cortical maturation using both EEG (specifically slow wave sleep activity (SWA)) and MRI data collected from 36 TD subjects aged between 8–19 years. Both sleep SWA and cortical gray matter volume decreased during adolescence, particularly in central and parietal regions of the brain. This study provided evidence that SWA during sleep is a good electrophysiological marker of cortical changes in adolescents [25]. This methodology could equally be employed to study volumetric changes in neurodevelopmental disorders like ASD and FXS, where MRI studies can be methodologically more challenging. This would facilitate better powered studies that are representative of a larger range of affected individuals which would further increase understanding of pathophysiology, help early diagnosis and guide potential therapies.

**Table 1:** A comparison of Fragile X Syndrome (FXS) and Autism Spectrum Disorder (ASD) through neuroanatomy and neuroimaging studies.

Study (year)	Disorder Studied	Method Employed	Subject Number (*n*)	Regions Investigated	Abnormality
Reiss (1988) [28]	FXS	MRI	FXS, 4 males.	Cerebellum	Significantly ↓ size of posterior portion of the cerebellar vermis.
Greco (2011) [29]	FXS	Post mortem neuropathological study.	FXS, 3 males.	Cerebellum and hippocampus (HC).	Significant morphological changes in both the cerebellum and HC.
Meguid *et al.*, (2012) [30]	FXS	MRI	FXS, 11 males.	Cortical morphology	↑ in whole hemispheric and lobar cortical volume.
Courchesne *et al.*, 2001 [31]	ASD	MRI	ASD 60 participants	Total Brain volume (TBV)	↑ TBV in 90% of ASD group 2–4 years.
Carper *et al.*, 2002 [32]	ASD	MRI	ASD, 38 males	Gray Matter Volume (GMV)	2–3 years, GM and White Matter (WM) hyperplasia (20% enlargement) no difference in 9–11.5 years.
Redcay & Courchesne 2005 [ 33]	ASD	Meta-analysis of 15 studies.	ASD, 49 males.	TBV	↓ in brain size at birth, dramatic ↑ within 1st year, plateau by adulthood.
Dalton *et al.*, 2008 [23]	ASD & FXS	MRI	9 FXS, 14 ASD, 15 controls.	Fusiform Gyrus (FG).	Both groups ↓ activation in FG associated with looking at faces. ↑ Activation in FXS compared with ASD and controls in general.
Hazlett *et al.*, 2009 [34]	FXS & ASD	MRI	FXS, 52 males, ASD 63 males, Developmental Delay (DD) 19 males, Controls 31 males.	Brain volume in substructures associated with behavioural features of ASD.	FXS + ASD had substantially enlarged CN volume and smaller amygdala (AMY) than FXS only. ASD subjects modest ↑ in CN volumes, compared to controls more robust ↑ in AMY volume.
Wilson, (2009) [35]	FXS & ASD.	MRI	FXS 10 (7 male), ASD 10 (8 male) and Controls 10 (7 male).	Voxel Based Morphometry (VBM) to identify volumetric changes.	Regional GMV in frontal, parietal, temporal and cingulate gyri as well as CN and CRB, were larger in FXS group relative to ASD.
Hoeft (2011) [36]	FXS & ASD	MRI	52 FXS, 63 ASD	Whole brain morphometric patterns.	Generally ↑ volume in ASD compared to controls, ASD in turn had ↑ volume compared to FXS.

## 2. Electroencephalography (EEG)

EEG records electrical activity on the scalp resulting from voltage fluctuations associated with ion flow within neurons. EEG benefits from excellent temporal resolution providing a near real-time neuroimaging signal time-locked to a specific task or sensory stimulus. It has the potential to generate diagnostic and treatment biomarkers to enhance clinical care [37]. This review examines all relevant literature, which includes all available FXS studies reporting EEG based analysis and the most relevant ASD EEG studies to date on this subject. In the following sections, each disorder will be addressed under the titles of Event Related Potentials (ERPs) and Resting State EEG (rEEG). Findings from both analysis methods will be compared in the discussion.

## 3. ERP Studies

Event Related Potentials (ERPs) are averaged EEG signals time-locked to specific sensory, cognitive or motor events (see Box 1). ERPs are commonly named due to their polarity (positive/negative) and time onset in milliseconds (Ms). The focus here is on ERPs that can be linked to behavioural symptoms or have potential as endophenotypes, which would indicate a relationship to the underlying mechanisms and the presence of symptoms. One of the most commonly investigated ERP components is the P300, which is associated with attention as well as decision making and novelty detection, all of which can be affected in ASD [38] and FXS [39]. The P300 can be broken into two subcomponents: the P3a and the P3b. The P3a is said to originate from stimulus-driven frontal attention mechanisms during task processing, whereas P3b is reported to originate from temporal-parietal activity associated with attention and appears related to subsequent memory processing [38]. Another ERP component that is commonly studied particularly in ASD research is the N170, which has long been associated with facial recognition (see Box 1).

## 4. ERP Studies in FXS

Based on the extant literature, less than ten relevant FXS studies based on ERPs exist. These studies include those who have investigated the novelty detection (P300) ERP component, as well as some earlier sensory components. In one of the earliest studies, St Clair *et al.* (1987), employed P300 components to investigate auditory response to an oddball paradigm in 33 adult subjects with FXS compared to age-matched typically developing (TD) controls. The oddball paradigm is widely employed, as it is associated with robust and reliable ERPs that are related markers of cognitive function [38]. The participant is typically required to respond to the target stimulus in order to assess attention and novelty detection. A typical oddball paradigm consists of two stimuli, one standard that occurs 80% of the time and one target which occurs 20% of the time.

St Clair *et al.* (1987), observed longer latencies and smaller amplitudes in all FXS subjects. This was interpreted as being associated with abnormal development of the hippocampus and related brain structures in the FXS subjects [40]. However Castren *et al.* (2003), in a much smaller study (*n* = 5), identified larger amplitudes of the N100 component to standard tones in FXS *versus* TD controls, also with an auditory oddball paradigm. Findings show maximum global field power (GFP) of the N200 component to be significantly larger in FXS. GFP is the standard deviation of the amplitude of the ERPs at all electrodes of an average reference. Subjects with FXS showed no habituation of N100 and no N200 sensitization for repeated tones. The authors suggested that these findings may indicate an increase in auditory sensitivity in subjects with FXS [41]. This is notable and in agreement with the studies reported on FXS mouse models which have also been observed to suffer from audiogenic seizures [42]. Furthermore, ERP abnormalities in response to an auditory oddball paradigm have also been described in male carriers of a premutation expansion of CGG repeats in the FMR1 with Fragile X-associated tremor/ataxia syndrome (FXTAS), a neurodegenerative disorder associated with Parkinsonism in addition to late onset tremor and ataxia. Prolonged latencies of N100 and P300 and reduced amplitudes of P200 and P300 were identified while the N200 ranges were observed to be normal. The smaller P300 amplitudes correlated with the increased length of the CGG repeat of FMR1. It is not clear whether these findings reflect later manifestations of earlier developmental changes in the brain, or occur as part of a later onset neurodegenerative process [43,44].

Box 1 **Most commonly reported Event Related Potentials (ERPs), where they occur and what they mean.**

**P100** = A Positive going component with peak occurring at approximately 100 ms post stimulus. The P100 reflects early sensory processing.
**P200** = A positive going potential that peaks around 200 ms post stimulus. P200 is found to be maximal over centro-frontal and parieto-occipital areas of the brain. Appears to be modulated by a diverse number of cognitive tasks.
**N100** = A large negative going evoked potential, it peaks in adults between 80–120 ms, post stimulus. Localized largely in the fronto-central region of the scalp. It occurs in response to sensory stimuli or unpredictable stimuli.
**N170** = Involved in the neural processing of faces. ERPS elicited from images of the face are compared to those elicited by other visual stimuli, the former show increased negativity 130–200 ms after stimulus presentation. N170 is found to be maximal over occipito-temporal electrode sites.
**N200** = A negative going wave that peaks between 200–350 ms post stimulus and is found primarily over anterior scalp sites. It is thought to be a mismatch detector, but has also been found to reflect executive cognitive functions. It has also been recently been used to study language.
**P200** = A positive going electrical potential that peaks between 150 and 275 ms, post stimulus. Located around centro-frontal and parieto-occipital regions. It is modulated by a large diverse number of cognitive tasks. P2 is typically elicited as part of the normal response to visual stimuli.
**P300** = A positive going component occurring between 250–500 ms. ERP component elicited in the process of decision making. P3 is thought to reflect processes involved in stimulus evaluation, novelty detection or categorization. Usually elicited by the oddball paradigm in which one stimulus occurs a small number of times (target) and another occurs the majority of the time (standard). Measured most strongly in the parietal lobe. The presence, magnitude, topography and timing of this signal are often used as a metric of cognitive function in decision making processes.
**Mismatch Negativity (MMN**) = An ERP component that occurs due to an odd stimulus in a sequence of stimuli. It can be elicited regardless of the individual’s attention. The localisation of the MMN can change depending of the nature of the stimulus.

A more recent investigation of selective attention in FXS (*n* = 16) used both auditory and visual experiments. Selective attention refers to the simple act of focusing on a specific object, while ignoring any irrelevant information that is occurring simultaneously. In this study, increased N100 and N200 amplitudes were observed, replicating the findings of Castren *et al.* (2003) [41]. A considerably attenuated auditory P3b response to deviant stimuli was also identified in FXS as previously described [39]. The attenuated P300 amplitude was less pronounced for visual compared to auditory stimuli. Other more recent studies have replicated this increased N100 amplitude in patients with FXS in comparison to controls [45,46,47,48]. Overall, increased amplitude of the N100 component appears to be the most consistently reported finding in FXS. The N100 amplitude is dependent on arousal and attention and is thought to be suggestive of hyperarousal in patients with FXS. The attenuated P300 may suggest attentional deficits in FXS.

## 5. ERP Studies in ASD

ASD has been more widely investigated using electrophysiological methods in comparison to FXS. Atypical P300 latencies have been reported in ASD participants in response to stimuli in different modalities and stimuli (speech, auditory visual and somatosensory) [49]. Atypical P300 amplitudes have also been reported [50]. Here the focus is largely on visual and auditory ERP studies, in particular the P300, as well as the N170 which as mentioned previously is associated with facial recognition. Face processing may be impaired in both ASD/ FXS in association with social cognitive deficits exhibited by both conditions.

Auditory ERPs can be elicited through the presentation of simple and complex auditory tones. They are commonly studied in ASD. Deficits have been observed in both high level and low level auditory processing in ASD [51], this review focuses on higher level cognitive processing. Courchesne *et al.* (1984) [46], found reduced amplitudes of two auditory P300 components to novel stimuli and reduced P3b amplitude to target stimuli in subjects with ASD in comparison to TD controls. The oddball paradigm consisted of the spoken word “me” as the target and mixes of human computer and mechanical sounds as the standard stimuli. The authors concluded that the ASD group, although processing the sounds correctly, may have been processing less efficiently than the TD group. In a more recent study, Ceponiene *et al.* (2003) [52] employed an auditory oddball paradigm consisting of three stimuli, simple tones, complex tones and vowel sounds, in a sample of nine High functioning Autistic (HFA) children and ten TD controls. P100 amplitudes tended to be smaller in children with ASD to standard stimuli although the differences were not significant. At a cognitive level, they observed no P3a component in children with ASD in response to vowel sounds, though this did not seem to be related to the complexity of the sound since the ASD group demonstrated a comparable response to controls and to frequency changes between sound stimuli in the Mismatch Negativity (MMN) paradigm (see Box 1). When the EEG data was combined with behavioural data, they concluded that the deficit may be due to the “speechness” quality of the sound, as children with ASD had been shown to exhibit deficits in orienting to complex social stimuli, whereas they exhibit no difference to TD peers in orienting to complex non-social stimuli. Another auditory study reported an increased occipital P300 to task relevant stimuli. Kemner *et al.* (1995) [53] hypothesised that this may relate to atypical sensitivity to auditory task related stimuli within the occipital lobe in ASD. A more recent study observed lower amplitudes in a number of components including the P100, N200 and P300 in a non-attended condition [54,55]. These studies suggest deficits in both early auditory processes and later cognitive processes, such as language processing and attention in ASD.

Visual ERPs have also been widely studied in ASD. Sokhadze *et al.* (2009) [49] found that ASD subjects exhibited longer P3a component latencies to novel stimuli. The authors suggested that this may be due to low selectivity in pre-processing and later stage under-activation of integrative regions in the prefrontal cortices [56]. The results of visual ERP studies in ASD are inconsistent. Pritchard *et al.* (1987) [57] found no difference in P300 amplitudes between ASD group and controls [50]; however, in a flash experiment, the ASD group showed a significant increase in N100 amplitude with increased flash intensity compared to a control group [57], which has been replicated in other studies [58,59]. Another study observed significantly reduced P100 amplitudes to biological and scrambled motion processing in ASD compared to TD controls. The TD group demonstrated right hemisphere lateralization of the N200 component in controls, which was not present in the ASD group. This lack of asymmetry was thought maybe to be related to an absence of development of specialized networks associated with deficits in visual and language processing in ASD [60].

Individuals with ASD have widely replicated deficits in emotional face processing. Consequently, the N170 component is one of the most commonly studied ERP components in ASD. Longer N170 latencies to faces have been observed in ASD relative to TD controls, despite comparable latencies to objects [23], this phenomenon has been replicated [61]. It has been demonstrated that, in general, the N170 is enhanced depending on visual directed attention in typically developing individuals; however, this was not observed in ASD subject groups. One study showed no modulation in N170 component between directed attention and non-directed attention. It was concluded that this may be due to reduced underlying neuronal activation [62]. This latter phenomenon parallels (at least for ASD) with reported decreased activation of the FG in ASD in response to faces, which is also observed in FXS group [63].

More specific aspects of face processing, such as discrimination between familiar and unfamiliar faces have also been investigated. One study showed higher N170 amplitudes in the left hemisphere to names and higher in the right hemisphere to faces in TD compared with ASD subjects and a higher P300 amplitude was observed in both groups in response to their own faces and faces of close others, for example faces of family and friends. However, the P300 amplitude did not differ significantly in the ASD group for their own faces. This contrasted with the TD group who had higher P300 amplitudes to their own face. This data may reflect differences in discrimination strategies [64]. This self-face recognition compared to familiar and unfamiliar faces was subsequently replicated; increased P300 amplitude to self-face was observed in the TD group. No significant differences in P300 components in response to self, familiar or unfamiliar faces were observed in the ASD group. This may reflect deficits in face encoding associated with atypical social communication [65]. Webb *et al.*, (2012) [63] assessed ERP responses to inverted faces, upright faces and houses. No differences in the ERP responses (namely increased P1 and N170 amplitudes) were observed in the faces *versus* houses paradigm in either group. The control group showed a significantly different N170 component to upright *versus* inverted faces, which was not observed in ASD [66]. These findings have been replicated in MRI studies, with studies observing reduced activation in ASD participants in areas such as the FG, amygdala, and the frontal cortex [67,68,69].

## 6. Resting State EEG (rEEG)

The use of EEG to observe the activity of the brain while at rest is a growing area of research in neurodevelopmental disorders, due to the availability of new analytical methods. rEEG data is obtained while the subject is “at rest”, *i.e.*, not performing any type of overt task and is presented with little or no sensory stimulation. In contrast to ERPs, which can be difficult to interpret without the fundamental knowledge of functional differences of the brain at rest, rEEG is accessible to a wider range of age groups and differing levels of intellectual ability as it is easy to administer and does not require the subject to be verbal. The absence of task-related movement also limits movement-related artefacts in the raw EEG data. One commonly employed method of analysis of rEEG is through the use of frequency bands. The typically investigated bands are: alpha (8–13 Hz), beta (13–30 Hz), delta (1–3 Hz), theta (4–7 Hz) and gamma (30–50 Hz). Simply, time dependent EEG signals can be decomposed into frequencies and the power spectrums analysed. Each specific frequency band represents a different cognitive state (See Box 2).

Box 2 **Most commonly reported EEG frequency bands and what they are thought to represent.**

**Alpha (8–13 Hz):** Usually occurs when a person is relaxed or in a trance like state.
**Beta (13–30 Hz):** Has been associated with states of alertness and agitation
**Delta (1–3 Hz):** Usually occur when a person is in a state of lethargy and are not attentive
**Theta (4–7 Hz):** Intuitive creative can also be unfocused.
**Gamma (30–50 Hz):** Thinking, integrated thought. High-level information.

## 7. Resting State in FXS

Similarly to ERP studies there are relatively few rEEG studies reported in FXS. Berry-Kravis *et al.* (2002) [64] identified increased epileptiform abnormalities, particularly centrotemporal spikes, in individuals with FXS both with and without seizures. More recently, subjects with FXS have been found to have elevated theta power, which has been hypothetically related to underlying glutamatergic activity [70]. Reduced relative upper-alpha power during an eyes closed resting state paradigm in temporal, frontal, central and parieto-occipital clusters has also been reported in FXS [71]. This finding was replicated in a subsequent study which also reported decreased global functional connectivity in FXS males in upper alpha and beta frequency bands and increased connectivity in long-range and short-range clusters in theta oscillations [72,73]. Cantor *et al.*, (1986) [71] concluded that these altered neural oscillatory dynamics may be associated with abnormal neuronal maturation in FXS, such as the uncontrolled synaptic overgrowth which has been demonstrated in FXS [1].

## 8. Resting State in ASD

Compared to ERP studies, rEEG has been more widely researched in ASD than in FXS. The greater delta and reduced alpha activity observed in FXS has also been observed in individuals with ASD and individuals with Intellectual Disability (ID), compared to controls with ID only. Additionally, less inter-hemispheric asymmetry has been observed compared with either TD children or children with ID [74,75]. This corresponds with observations made with both diffusion spectrum imaging tractography and MRI [76,77]. Other studies have shown epileptiform discharges at rest in 20% of cases with ASD, without the presence of clinical seizures [78].

Murias *et al.* (2007) [79] found reductions in long-range alpha band coherence reductions (frontal-occipital, frontal parietal) in adults with ASD, suggesting weak functional connections between the frontal lobe and the rest of the cortex [80]. This idea of coherence refers to a way to measure functional interactions between oscillating brain sub-systems. This result has been replicated in several other studies [81] and reflects expanding knowledge regarding altered functional connectivity between brain areas in ASD [82,83,84,85]. Coben *et al.* (2008) [78] observed excessive theta activity, primarily in right posterior regions in children with autism. In contrast with other studies, they found a pattern of deficient delta over the frontal cortex and excessive midline beta. Additionally, they identified a pattern of under connectivity in the ASD group using indices of absolute and relative power for four frequency bands (delta, alpha, beta and theta). Intrahemispheric coherence mathematical means were compared across short/medium and long inter-electrode distances [78]. Delta and theta power have been shown to be inversely related to activations of the default mode network (DMN). The DMN is a network of brain areas that are active when the individual is not focused on the surrounding environment and the brain is at wakeful rest [86]. Other neuroimaging studies have observed atypicalities in the functional connectivity of the DMN in participants with ASD [87,88,89].

Although not widely investigated, gamma band power has been implicated in inhibitory interneuron function and is thought to represent GABA concentration [90,91]. GABA interneurons have been implicated in the neurobiology of both FXS [70] and ASD [92]. One group compared developmental changes in rEEG power in two groups of young children aged 6 and 24 months, defined by high and low risk for ASD. The high-risk group were shown to exhibit lower spectral power in all frequency bands at 6 months of age [90]. Spectral differences largely normalized with development, with the exception of alpha and gamma bands in the high-risk group. Gamma band power has also been observed to be negatively associated with language skills and intellectual abilities in children with a family history of impaired speech [63]. Studies using other imaging modalities, for example magnetoencephalography (MEG), found largely absent gamma response to faces in the occipital areas of the brain in subjects with ASD [93].

## 9. Methods: Selection of Stimuli, Numbers and Power Issues

The majority of studies examined in this review have focused on ERP studies. ERPs are extracted through averaging of EEG responses to specific cognitive or sensory events. ERPs provide a fine temporal resolution of brain activity up to and after a specific event. ERP analysis generally focuses on peak amplitude and latencies of components of interest as well as over scalp Regions of Interest (ROI). For example, the analysis of the oddball paradigm frequently focuses on activity over temporal and central regions of scalp. As described, ERP studies of FXS have focused almost solely on the oddball paradigm (Table 1) using simple auditory [39,41,94] or visual stimuli [42]. The auditory paradigms were either passive or required a response.

FXS is a relatively rare disorder (1–4 in 6000) and varies significantly in severity which makes recruitment of participants difficult. Many FXS EEG studies are underpowered, with most having fewer than 20 subjects. While ASD studies have typically used larger sample sizes, clinical heterogeneity will also impact power and interpretation of findings. ERP studies with ASD participants typically focus on those that are cognitively more able with average to high IQs, who typically can follow task instructions and sit still for the required time. ASD children with a lower cognitive function are likely to also present assessment challenges relating to sustaining attention, hyperactivity and behavioural issues. However 50% of individuals with ASD exhibit some level of ID and therefore there is a need to study individuals with greater cognitive challenges to understand brain function more broadly in ASD. Key questions arise therefore in relation to sample size, power and tolerability. Firstly, how can sample sizes and, consequently, power be improved? Secondly, do we need new data collection strategies and experimental paradigms that are accessible to participants with neurodevelopmental disorders and greater cognitive challenges? The solutions to both of these issues are possibly related since improving tolerability may improve participation in research. Conventional mouse and desktop set ups for experimental paradigms may induce anxiety, particularly in neurodevelopmental disorders such as FXS and ASD that are associated with a range of socio-emotional and behavioural challenges. Although resting state data may be the easiest to collect in a research environment, the results from our review show that this global measurement indicates little difference between FXS and ASD and may not be sufficiently discriminating or specific. None of the studies, however, included both FXS and ASD individuals. Cross disorder studies using the same methods will give a more accurate picture of the rEEG differences and similarities between ASD and FXS.

As mentioned previously, both ASD and FXS are more prominent in males than in females. This obvious gender difference exhibited in both FXS and ASD is not well represented in the available literature in this area. Of the FXS EEG studies mentioned in this review, only one has specified that they used female participants with FXS [43]. This bias towards male subjects may also be a confound of this type of research, as females with FXS are less likely to have severe IDs than males with FXS due to the compensatory effect of a second X chromosome. Future studies into these gender differences as well as female specific studies may lead to the formation of a greater overall picture of these complex disorders.

Confounds in clinical study design may impact on accurate data interpretation. Many ASD EEG studies reviewed in the literature did not match subjects and controls for IQ. Clear differences in ERPs are observed in subjects with ASD in relation to IQ differences, as demonstrated by Salmond *et al.* (2007), who detected longer P3a latencies to novelty and lower P3b amplitude in a group with lower verbal IQ (VIQ) *vs.* a group with higher VIQ, suggesting differences in attention-dependent novelty processing are linked to cognitive impairment in ASD [95]. IQ was not highlighted as a variable in some of the FXS studies reviewed [41]. Some studies mentioned, did not state that both the verbal and non-verbal IQ of FXS participants were significantly lower than the control group [42,65,66,94]. Discrepancies in IQ, as stated before, should be addressed in future studies.

Technical considerations are also relevant. The most recent FXS study reviewed here employed an array of 26 EEG electrodes [66]. The use of higher EEG electrode densities, particularly in FXS, may provide more detailed information than previously collected on connectivity and coherence. While the EEG acquisition process can be better tolerated by children with intellectual disabilities than other neuroimaging methods, sensory issues and deficits in attention can be problematic, and the increased set up time associated with higher EEG electrode densities may have an impact on study feasibility. Therefore, alternative methods of acquiring high-density EEG data for these types of participants, such as the use of dry electrodes should be explored.

Recruitment is consistently an issue in clinical research, particularly for neurodevelopmental disorders like ASD and FXS where high career burden and stress can impact on the ability to commit time to research, particularly in the absence of a tangible, direct benefit. Since sample size and power are significant considerations, data sharing is a practical way to address this issue, particularly with EEG data where there are less methodological differences between acquisition methods, and activity at specific electrodes can be compared across cohorts. This will require collaborative research and standardised protocols and has been previously undertaken successfully with MRI data in ASD [96]. Additionally, there is a need for a testing environment that is sympathetic to the needs and potential anxiety of subjects and, therefore, greater use of interactive games that promote natural behaviour, may be more acceptable, less anxiety provoking, and lead to increased participation.

Finally, genetic testing is increasingly being made available to those with neurodevelopmental disorders. This is of paramount importance to the type of research reviewed in this paper. Due to the small number of EEG studies in this area, it is not practical to exclude all studies where no genetic testing has been carried out on all participants. However, for future work, this is essential in order to gain a true understanding of the differences and similarities between these disorders. Moreover, EEG studies might similarly become more relevant in the investigation of newly defined genetic ASD syndromes, e.g., deletion/duplications of 16p11.2, SHANK3 mutations, NRXN1 deletions that are increasingly being identified by whole genome studies in ASD.

## 10. Discussion

This review has focused on the available, relevant literature on EEG abnormalities in two neurodevelopmental disorders with suggested genetic, molecular and behavioural overlaps. These disorders have shown some similarities/differences which may suggest that some of these mechanisms may be reflected in EEG changes. Therefore, EEG signatures may provide the basis for endophenotypes to study these conditions. However, there are limitations in the studies reported to date that need to be addressed in future research.

## 11. Interpretation of ERPs Studies in ASD and FXS

The main focus of this review has been on EEG-derived ERPs that represent higher level cognitive and social processing. The reason for this focus specifically on ERP components is to probe overlapping behavioural symptoms of ASD and FXS through electrophysiological findings. Both ASD and FXS have been associated with deficits in attention. The P300 component is associated with attention and change/novelty detection. Attenuated P300 components have been observed in both FXS and ASD in both the auditory and visual modalities. This may suggest that there are some similarities in information processing deficits in these two neurodevelopmental disorders. These diminished amplitudes may suggest less synchronicity in neuronal activation in response to sensory stimulation in ASD and FXS subjects in comparison to control subjects. Conversely, an increased N100 response to sensory stimuli has been reported in several FXS studies, which appears to be specific to the FXS population, suggesting that there are morphological differences between ASD and FXS, e.g., brain regions associated with the generation of an N100 component or neuronal activation may be less inhibited in FXS. Dawson *et al.* (1988) [63], reported longer duration P300 latencies and lower P300 amplitudes, in an auditory paradigm involving phonetic speech in ASD [48]. This attenuated amplitude has also been reported in auditory experiments involving subjects with FXS. There are several explanations, which may help to explain why subjects with ASD and FXS show differences in ERPs. One is that novelty is processed in a different way in ASD. It may be that ASD groups used abnormal or alternate processes to detect targets in an oddball paradigm [46,47]. Another suggests that diminished P300 amplitudes may be due to abnormalities in underlying mechanisms of selective attention, which may, in turn, underlie the cognitive deficits in ASD [97]. The brain areas often associated with P300 generation are the parietal and frontal cortices. The parietal lobe is often associated with the integration of sensory stimuli and, therefore, abnormal function/development of this region may lead to problems in sensory integration which could lead to a cascade of other problems, such as the social and communication deficits associated with both ASD and FXS. Finally, the reduced P300 amplitudes may suggest that participants with ASD recruit less attentional resources into the further cognitive processing of sensory stimuli. While it is clear that there are differences associated with processing sensory stimuli in both ASD and FXS compared to TD controls, it remains to be seen whether these relate to deficits in attention. The similarities in P300 components in ASD and FXS is an area for further investigation and may provide insight into underlying neural mechanisms of these disorders.

One of the areas where both individuals with ASD and FXS show major deficits is in social communication and interaction. These social deficits explain why the N170 component is so highly studied in neurodevelopmental disorders and, particularly, in ASD. The N170 has been consistently associated with facial recognition. As with the P300 component, many different conclusions have been drawn from altered visual N170 components in ASD. Longer latencies of the N170 component have been reported to be due to slower processing of faces in ASD [98]. It has also been suggested that individuals with ASD have increased sensitivity to configural properties of faces or the structural perception of faces but not personal identity [62]. The evidence from these studies may suggest that recognizing that a face is a face may occur through low level first order processing of facial features (eyes, mouth, nose), whereas recognizing that the face is different (inverted, familiar) may have to do with second order processing. This may explain differences observed in the N170 [66,78]. This theory conflicts with other ASD studies that have reported that individuals with ASD direct their gaze to the periphery of the face rather than concentrating on the facial features. However, the idea that children with ASD are more sensitive to the facial features does not align with other research that reports individuals with ASDs concentrate more on details in comparison to global features of an object [99]. From the evidence above, it is clear there are deficits in facial processing associated with ASD but where exactly these deficits occur is uncertain.

Although there are no specific EEG studies in FXS studying face recognition, there have been studies that have focused on eye gaze in FXS, and it may be possible to relate EEG findings in ASD to MRI findings in FXS. One such MRI study employed photographs of forward facing and angled faces, each having direct or averted gazes. Eleven female subjects and eleven controls were asked to determine the gaze direction for each photograph. Areas of the brain often associated with face and gaze stimuli, the fusiform gyrus (FG) and the superior temporal sulcus (STS), were compared between the two groups. The FXS group showed decreased accuracy in determining the gaze direction compared to controls. The control group showed decreased activation to angled faces in comparison to straight on faces, whereas the FXS group showed no difference in FG activation to angled faces in comparison to straight on. The group suggested that gaze aversion in FXS is related to decreased specialization of the FG in the perception of face orienting and that this, in turn, may suggest dysfunction of neural systems underlying both face and gaze processing [100]. Clearly, given the paucity of research, more investigation is required to be conclusive.

Both ASD and FXS subjects have shown reduced P3 amplitudes to novelty detection in comparison to typically developing individuals. However, only the FXS group showed a consistent increase in the N100 amplitudes to sensory stimuli. This may suggest that early sensory processes in FXS and ASD are different, despite potentially similar mechanisms relating to higher level cognitive processes. By identifying these differences, it may be possible to distinguish these disorders from each other and TD controls using electrophysiological markers. Recently, Brandwein *et al.* (2014) identified a relationship between electrophysiological indices of auditory processing and autism symptom severity [101]. This demonstrated the potential of this type of research for the future of neurodevelopmental disorders.

## 12. Resting State in FXS and ASD

Based on the extant literature, similar trends have been observed in studies using rEEG data in both FXS and ASD. Studies in both ASD and FXS subjects have revealed an increase in theta activity and a decrease in alpha activity across these two groups. Analysis of rEEG data also suggests a pattern of neural underconnectivity in both conditions [102]. The exact function of theta activity in neurodevelopmental disorders is not fully understood, however increased theta power has been associated with ADHD [2,103] and learning disabilities in children [104,105]. Other studies in ASD have linked rEEG data to cognitive function, e.g., language and intellectual ability, and correlate inversely with activation in the DMN [106]. These are avenues that may also be explored in FXS given adequate sample sizes. Functional MRI has demonstrated a failure to deactivate in these DMN regions in ASD subjects during a task. This may have to do with the absence of mental processes that normally occur during rest [107]. This failure to deactivate resting state networks has also been demonstrated to some extent in FXS [108], possibly suggesting an abnormality in inhibitory mechanisms in both FXS and ASD leading to global deficits. These similarities suggest deficits in resting state connectivity in the two disorders in comparison to TD controls.

In order to fully understand neurodevelopmental disorders such as FXS and ASD, information needs to be gathered at critical developmental periods. This is particularly relevant to brain volume where significant developmental differences have been observed in FXS and ASD, relative to each other in addition to TD children. Rapid growth and overall larger total brain volume are observed in both ASD and FXS compared with TD [24,36]. These brain volume differences have been observed to plateau around adolescence, showing the importance of studies of brain structure and function during childhood. The vast changes the brain goes through during these critical stages of development may have a large impact on the validity of data, particularly in studies using wide age ranges. Longitudinal studies are critical to the understanding of atypical neurodevelopment although they are expensive and resource intensive. Some recent studies have attempted this longitudinal design [109]. Analysing the brain at different stages of development, however, is likely to be more informative regarding the underlying mechanism of these disorders. Identifying abnormal trajectories that can distinguish neurodevelopmental disorders, such as FXS and ASD, from each other and from normal controls is likely also to be useful in the development of diagnostic and treatment biomarkers.

## 13. Relationship between EEG and Underlying Neurobiology: How Might EEG Aid in the Understanding of These Disorders Aetiologically?

Relating EEG data to underlying neurobiology in most studies has been speculative, though there is mounting evidence that neurophysiological abnormalities, particularly in rEEG, may be partly related to an excitation/inhibition imbalance in the ASD brain [110]. This imbalance has also been reported in the FXS literature [111]. Normal neural circuit function requires precise and efficient excitatory and inhibitory transmission in order to function properly, and any abnormalities in these circuits could cause a cascade of events leading to abnormal brain function.

FXS was the first described disease of synaptic plasticity, with both excitatory (e.g., mGluR) and inhibitory (GABA) mechanisms implicated here [112]. This leads to “excitation-inhibition” imbalance as a possible underlying mechanism for FXS [113]. Absent or reduced FMRP expression in FXS has been linked to imbalanced cortical excitatory (glutamatergic) and inhibitory (GABAergic) circuit activity in FMR1 knockout mice [114,115,116]. Additionally, over activation of metabotropic glutamate receptor 5 (mGluR5), a metabotropic glutamate receptor, has been linked to deficits in synaptic morphology and plasticity. Compounds that reduce excess glutamate signalling have been shown to improve behavioural and morphological deficits associated with FXS in animal models [117]. GABA agonists such as Arbaclofen have also shown demonstrated potential for reduction of social function and behaviour in FXS [118].

The molecular mechanisms for ASD are far less clear than for FXS; however, several lines of evidence point to this excitatory/inhibitory imbalance also being a mechanism for ASD [119]. Single nucleotide variants and copy number variants (CNVs) in genes implicated at glutamatergic synapses are thought to be pathogenic in small numbers of individuals with ASD. Common genetic variants that increase susceptibility for ASD have been shown to converge on FMRP molecular targets [120]. The dysfunction of inhibitory neurotransmitter GABA has been investigated extensively as a contributor to the phenotype of ASD, studies have shown alterations in mRNA encoding of enzymes GAD65 [8,121] and GAD67 [122,123] that decarboxylate glutamate to GABA in the brain, and alterations in GABA receptor levels [90]. Lower levels of FMRP and the GABA (A) receptor beta 3 proteins have been identified in the cerebellar vermis of adults with ASD in post-mortem (PM) brain studies. PM studies have shown significantly elevated levels of mGluR5 in the vermis of children with ASD. Neuroanatomical abnormalities in the cerebellar vermis have been described in both ASD and FXS [124,125]. Animal knockout models of genes implicated in excitation and inhibition are associated with ASD-like cognitive and social deficits. Consequently, the targeting of molecules to redress excitation-inhibition imbalance may provide avenues for new therapeutic development for both FXS and ASD.

EEG as a modality may provide an endophenotype reflecting excitation-inhibition imbalance and additionally serve as a biomarker for therapeutic trials. FXS patients are characterized by a high incidence of hyperexcitable (excessive reaction to stimulus) EEG patterns that is likely to relate to increased glutamatergic excitation [105]. Abnormalities in rEEG are hypothesised to be partly attributable to abnormal functioning of (GABA)ergic tone in inhibitory circuitry in ASD and are likely to influence the functional and developmental plasticity of the brain. Spectral analysis of rEEG data has shown atypicalities in both ASD [78] and FXS [65,66] in alpha and theta activity levels. One study suggests exaggerated glutamatergic activity may be the cause of these theta frequency oscillations. As a result of immature cortical networks in the brain of FXS, this may also be the case with ASD subjects [51].

How EEG and the underlying neurobiology are linked together is extremely important for future research. The question has to be asked: How would an imbalance in neural excitation and inhibition lead to abnormalities in EEG data? As reported here, the literature shows that a cascade of processing difficulties occur in both FXS and ASD. One theory that has been put forward is contextual modulation (CM). CM is the automatic compensation of the visual system for the blind spot by creating a contiguous pattern over this blind spot. Some scientists believe that this excitatory/inhibitory imbalance may give rise to this atypical CM leading to the atypical visual perception [114]. Only two ERP studies have reported the visual modality in FXS and both demonstrated higher N100 and N200 to visual stimuli in FXS [43]. One study saw attenuation of the N100 waveform in children with FXS after three months of treatment with minocycline. Minocycline has been shown to normalise synaptic connection in knockout mouse models of FXS [126]. Schneider *et al.* (2013) [127] believed that the attenuation of the N100 may have been due to reduced auditory excitability with minocycline. This study elucidated the potential for EEG as a tool for assessing treatment endpoints in neurodevelopmental disorders [127]. A combination of neurophysiological and behavioural data with what is known about the neurobiology of these disorders may lead to the development of new methods for diagnosis of ASD and FXS, as well as providing further insight into the underlying mechanisms and creating drug treatment endpoints.

## 14. Conclusions

In conclusion, it can be appreciated that the clinical and scientific need to probe deeper into disorders such as ASD and FXS has never been greater. The questions arising from the review of the literature in this domain include:

1. How can topology of the neuronal networks be connected to the underlying neuronal mechanisms of ASD and FXS through electrophysiological measures, in order to gain a greater understanding of the underlying neurobiological processes of these disorders?
2. Can EEG and EEG-derived data be employed as a method to assess the similarities and differences in neural processing between FXS and ASD and TD controls, in order to be able to distinguish between these groups?
3. Can biological markers be identified that are unique to each group, so that earlier diagnosis of these disorders may be possible, and thus possibly lead to earlier intervention?
4. Is it possible to recruit larger subject groups with varying IQs, gender and symptom severity along with appropriately matched controls to inform on the developmental trajectories of these disorders?
